# Embryonic stem cell-derived extracellular vesicles promote the recovery of kidney injury

**DOI:** 10.1186/s13287-021-02460-0

**Published:** 2021-07-02

**Authors:** Lu Yu, Siying Liu, Chen Wang, Chuanyu Zhang, Yajie Wen, Kaiyue Zhang, Shang Chen, Haoyan Huang, Yue Liu, Lingling Wu, Zhongchao Han, Xiangmei Chen, Zongjin Li, Na Liu

**Affiliations:** 1 School of Medicine, Nankai University, Tianjin, 300071 China; 2 Key Laboratory of Bioactive Materials, Ministry of Education, College of Life Sciences, Nankai University, Tianjin, 300071 China; 3 State Key Laboratory of Kidney Diseases, Chinese PLA General Hospital, Beijing, China; 4 Beijing Engineering Laboratory of Perinatal Stem Cells, Beijing Institute of Health and Stem Cells, Health & Biotech Co., Beijing, China

**Keywords:** Embryonic stem cell (ESC), Extracellular vesicles (EVs), Acute kidney injury (AKI), Physiological repair, Pathological repair

## Abstract

**Background:**

Embryonic stem cell-derived extracellular vesicles (ESC-EVs) possess therapeutic potential for a variety of diseases and are considered as an alternative of ES cells. Acute kidney injury (AKI) is a common acute and severe disease in clinical practice, which seriously threatens human life and health. However, the roles and mechanisms of ESC-EVs on AKI remain unclear.

**Methods:**

In this study, we evaluated the effects of ESC-EVs on physiological repair and pathological repair using murine ischemia-reperfusion injury-induced AKI model, the potential mechanisms of which were next investigated. EVs were isolated from ESCs and EVs derived from mouse fibroblasts as therapeutic controls. We then investigated whether ESC-EVs can restore the structure and function of the damaged kidney by promoting physiological repair and inhibiting the pathological repair process after AKI in vivo and in vitro.

**Results:**

We found that ESC-EVs significantly promoted the recovery of the structure and function of the damaged kidney. ESC-EVs increased the proliferation of renal tubular epithelial cells, facilitated renal angiogenesis, inhibited the progression of renal fibrosis, and rescued DNA damage caused by ischemia and reperfusion after AKI. Finally, we found that ESC-EVs play a therapeutic effect by activating Sox9^+^ cells.

**Conclusions:**

ESC-EVs significantly promote the physiological repair and inhibit the pathological repair after AKI, enabling restoration of the structure and function of the damaged kidney. This strategy might emerge as a novel therapeutic strategy for ESC clinical application.

**Supplementary Information:**

The online version contains supplementary material available at 10.1186/s13287-021-02460-0.

## Introduction

Acute kidney injury (AKI) is a syndrome characterized by rapid loss of renal excretory function that happens within a few hours or a few days, which is often associated with a variety of short- and long-term complications [1, 2]. Without prompt treatment, AKI can progress to chronic kidney disease (CKD), end-stage renal disease (ESRD), or even to death as it can lead to the formation of fluid and waste in the body, which is life-threatening [3, 4]. A large sample of epidemiological surveys abroad shows that the incidence of AKI among inpatients in an intensive care unit (ICU) is 26.9%, and the case fatality rate could reach 11.0% [5]. It is estimated that 2 million people worldwide die from AKI every year, and the incidence is rising [6]. AKI threatens the health and life of patients for its higher fatality and expensive treatment, and also causes a certain degree of consumption of medical resources. Therefore, it is extremely urgent to find a new and effective treatment for AKI.

Current treatments are mostly supportive therapies and do not directly target the disease. Regenerative medicine-based treatment might treat AKI directly. Several types of stem cells and progenitor cells have been reported as seed cells for regenerative medicine against kidney diseases. In mice with AKI induced by cisplatin, MSC inhibited apoptosis and promoted proliferation of renal tubular epithelium by decreasing the expression of microRNA-146b. Zhu et al. found that the level of microRNA-146b in renal tissues of rats with AKI induced by cisplatin was significantly upregulated. MicroRNA-146b may be an early biomarker of AKI and inhibition of microRNA-146b may be a new strategy for the treatment of AKI [7]. Renal progenitors derived from human-induced pluripotent stem cells (hiPSC) have also been reported to be therapeutic in a mouse model of AKI, alleviating histopathological changes in the damaged kidney [8].

Embryonic stem cells (ESCs) isolated from mammalian blastocysts possess multiple differentiation potentials which have gained significant attention in the field of regenerative medicine. ESCs can be induced to differentiate into all types of cells under certain conditions [9, 10]. It has been reported that ESCs have obvious therapeutic effects in the treatment of several diseases, such as neurodegenerative diseases, diabetes, necrotic myocardium, and liver regeneration [11, 12]. However, due to issues such as immune rejection, tumorigenicity, and ethics, the application of ESCs in clinical treatment is limited [13]. To avoid the ethics issue, several alternative sources have been reported, such as therapeutic cloning [14, 15], parthenogenetic ES cells [16, 17], and induced pluripotent stem cells (iPSCs) [18, 19]. However, their tumorigenic potential also limit the clinical application of pluripotent stem cells [20, 21].

Extracellular vesicles (EVs) are lipid membrane-delimited vesicles that are naturally secreted from many eukaryotic cells [22]. They play an important role in intracellular and intercellular communication [23], regulating of immune response, cell proliferation, cell migration, vessel formation, cancer progression, etc. [24]. The transportation and transfer of EVs will affect various physiological functions of their target cells, and even their pathological functions [24]. The role of EVs depends on the delivery of their contents to recipient cells, which in turn alters the biological processes of recipient cells [25]. EVs contain RNA (mRNA and miRNA), lipids (cholesterol, spines, ceramides, phospholipids, and dextran), and proteins from source cells and have certain characteristics of the cell from which it is derived [26]. In recent years, the focus of stem cell therapy has shifted from the stem cell itself to the stem cell-derived EVs. Compared with the whole stem cell, stem cell-derived EVs have some advantages. Owing to its natural origin, EVs have high biocompatibility and limited immunogenicity [27]. ESC-EVs are less tumorigenic than ES cells [28]. As an important way of paracrine activity, EVs play an important role in tissue regeneration [29]. ESC-EVs can avoid ethics problems because ESC-EVs are derived from the supernatant of ESC, and ESC can proliferate indefinitely in vitro, so we can continuously isolate ESC-EVs without destroying new embryos [30].

Several studies have reported that ESC-derived EVs (ESC-EVs) play an important role in the treatment of cardiovascular diseases [31]. ESC-EVs also promote the proliferation of mouse primary skin fibroblasts [32] and rejuvenation of senescent MSCs [33]. ESC-EVs are considered as a good substitute for ESCs in cell therapy, avoiding the defects of ESCs in clinical applications and greatly exerting the excellent characteristics of ESCs. However, the functions of ESC-EVs in the treatment of AKI are still unclear. Here, we explore the roles of ESC-EVs on AKI. Our data suggested that ESC-EVs promoted the repair of injured kidney after AKI by promoting physiological repair and inhibiting the pathological repair process.

## Materials and methods

### Cell culture

We used a commercial ESCs line (D3 ES cells), which was purchased from ATCC (CRL-1934). The mouse ESCs were grown on plates pre-coated with 0.1% gelatin and cultured in DMEM medium (Hyclone) supplemented with 15% FBS (Hyclone), 1% L-Glutamine (Corning), 1% NEAA (Gibco), 1% penicillin and streptomycin (Gibco), 1%β-mercaptoethanol (Sigma), and 1000 units/mL of LIF (Millipore). The mouse embryonic fibroblasts (MEF) were cultured in DMEM medium (Hyclone) supplemented with 10% FBS (Hyclone), 1% L-Glutamine (Corning) and 1% penicillin and streptomycin (Gibco). All FBS mentioned above were EV-free FBS. To obtain EV-free FBS, we performed ultracentrifugation at 120,000 g, 4 °C for 2 h. Then, the supernatant was carefully taken and the supernatant was filtered with a 0.22-μm filter membrane (Millipore). Human proximal tubular (HK-2) cells were purchased from ATCC (CRL-2190). The HK-2 cells were cultured in DMEM (Hyclone) with 10% FBS and 1% penicillin and streptomycin. Human umbilical vein endothelial cells (HUVEC) were purchased from ATCC (CRL-1730). The HUVEC were cultured with an EGM-2 medium (Lonza, Walkersville, MD). All cells were cultured at 37 °C in a humidified 5% CO_2_, 95% air incubator.

### Cell viability assay

To evaluate the optimal concentration of EVs to promote HK-2 and HUVEC proliferation, a cell counting kit 8 (CCK-8) assay was performed. In brief, cells were seeded in 96-well plates (2 × 10^3^/well) and treated with different concentrations of EVs for 48 h. Then 10 μl CCK-8 (TargetMol, MA, USA) solution was added to each well and incubated at 37 °C for 2 h. The optical density (OD) values at 450 nm were read using a microplate analyzer (Promega, WI, USA).

### Extracellular vesicle isolation

Extracellular vesicles were purified from supernatants of ESC and MEF as previously described [34–36]. When the fusion rate reached 70–80%, the cells were passaged and cultured in DMEM with EVs-free serum. After 24 h, the supernatant of cell culture was collected and centrifuged at 500*g*, 4 °C for 10 min to remove any dead cells. Then the cell debris and apoptotic bodies were discarded by a centrifugation step of 2000*g* for 30 min. ESC-EVs and MEF-EVs were isolated by ultracentrifugation at 120,000*g* for 2 h at 4 °C.

### Extracellular vesicle characterization

The morphology of EVs was observed by transmission electron microscopy (TEM) (Talos F200C, Thermo Fisher, MA). The samples were deposited on a copper grid covered with carbon film (Zhongjingkeyi Technology, Beijing, China) and dried at room temperature for 2 min. After removing the excess liquid with a filter, the samples were negatively stained with 2% uranium acetate for the 30 s. The samples were air-dried for 60 min and then imaged using TEM. The size of EVs was determined using nanoparticle tracking analysis (NTA) (Particle Metrix, Germany). A BCA Protein Assay Kit (Genstar, Beijing, China) was used to measure the protein concentration in EVs.

### Extracellular vesicle internalization

To verify whether EVs could be internalized by HK-2 and HUVECs, EVs were labeled using CM-DiI membrane dye (Invitrogen, Carlsbad, CA) according to the manufacturer’s protocol. EVs were mixed with 1 μmol /L CM-DiI and incubated at room temperature for 5 min. The excess dye was removed by ultracentrifugation at 100,000*g*, 4 °C for 70 min. The final labeled EVs were resuspended in PBS. CM-DiI-labeled EVs were incubated with HK-2 or HUVECs at 37 °C for 6 h, after washing the cells with PBS, the cells were fixed with 4% paraformaldehyde. The uptake of EVs was observed by fluorescence microscopy.

### AKI model

We established the ischemia-reperfusion-induced AKI model as previously described [37]. Briefly, male C57BL/6 mice (8–10 weeks old, weighing 20–25 g) were fed under proper ventilation conditions at a temperature of 21 ± 2 °C and a humidity of 60 ± 5%, maintained with a 12-h light-dark cycle, and freely took in food and water [38–40]. The mice were randomly divided into Sham group, PBS group, MEF-EVs, and ESC-EV treatment group. They were anesthetized with 2.5% avertin (Sigma-Aldrich) at a dose of 240 mg/kg by intraperitoneal injection. Under aseptic conditions, the left skin and peritoneum of mice were cut off, and the left renal pedicle was clamped with a noninvasive microvascular clamp for 40 min, the contralateral nephrectomy was performed. If the kidney immediately turned purple, the clamping was successful, and the kidney was kept warm and moist during this period. After removing the non-traumatic microvascular clamp, the kidney immediately begins to reperfusion. After 5 min of reperfusion, 100 μg EVs suspended in PBS were injected into the upper and lower poles of the left renal cortex, and PBS was injected into the same volume as the control (n = 5 in each group). Sham-operated mice underwent a similar surgical intervention except for the clamping of the renal pedicles. Similar skin and peritoneum incisions and sutures were performed in Sham group animals, but no IR injuries or injections were performed. Mice sampled at days 1, 3, 5, 7, and 14 after AKI were subjected to the bilateral renal injury model described above, with left kidney clipping and right nephrectomy. In order to improve the survival of mice sampled at day 28 after AKI, we used a unilateral kidney injury model with left kidney clipping and left right kidney untreated.

### Renal function analysis

For renal function analysis, eyeball blood was collected on days 1, 3, and 7 after injury, and the supernatant was obtained after centrifugation at 3000 rpm for 10 min to obtain mouse serum. Blood serum was collected to assess the concentration of BUN and SCr using the BUN assay kit (C013-1, Jiancheng Bioengineering, Nanjing) and the SCr assay kit (C011, Jiancheng Bioengineering Research Institute, Nanjing).

### Histological analysis of renal tissues

At indicated time points, the mice were anesthetized and perfused with ice-cold PBS to obtain kidney samples. For paraffin sections, kidney samples were fixed overnight with 4% paraformaldehyde (PFA) at 4 °C, dehydrated with ethanol, hyalinized with xylene, and finally embedded in paraffin (Leica Microsystems, Wetzlar, Germany). For cryosections, kidney samples were fixed with 4% paraformaldehyde and then dehydrated with 30% sucrose solution, and eventually embedded into Optimal Cutting Temperature Compound (Sakura Finetek, Tokyo, Japan). All samples were cut into sections with a thickness of 5 μm. The H&E staining, Masson staining, and immunohistochemistry were performed on paraffin sections, whereas cryosections were used for immunofluorescent staining.

For immunostaining, the cryosections were washed in 0.1% PBST, blocked in 10% goat serum for 1 h and incubated with a series of primary antibodies: Kim-1 (1:200; Abcam), α-SMA (1:200; Abcam), CD31 (1:200; BD Pharmingen), Ki67 (1:200; Abcam), γ-H2AX (1:500; Cell Signaling Technology), Sox9 (1:400; Cell Signaling Technology), and Tubulin (1:500; Proteintech) overnight at 4 °C. Then, the cryosections were incubated with secondary antibodies Alexa Fluor 488- and Alexa Fluor 594-labeled goat anti-rabbit or goat anti-mouse antibodies (1:500; Proteintech) at room temperature for 1 h. The FITC-labeled Lotus tetragonolobus lectin (LTL; 1:400, Vector Laboratories, Burlingame, CA) was used to disclose renal structure, and 49-6-diamidino-2-phenylindole (DAPI; 1:2000, Sigma) was used to visualize the nucleus. The quantitative analysis of the immunostaining was measured by ImageJ software.

### Western blotting analysis

Cells were lysed in RIPA buffer (Solarbio, Shanghai, China) in the presence of a 1 mM protease inhibitor (Solarbio, Shanghai, China) at 4 °C for 30 min. Subsequently, the sample was centrifuged at 4 °C, 14,000 rpm for 15 min, then the supernatant was collected. Protein concentration was determined using BCA Protein Analysis Kit (Genstar, Beijing, China) according to the manufacturer’s instructions. Finally, the samples were boiled at 95 °C for 10 min with 1× SDS sample buffer. The obtained protein samples were separated by 10% SDS-PAGE and then transferred to polyvinylidene difluoride (PVDF) membrane (Millipore, Darmstadt, Germany). After blocking with 5% nonfat milk for 2 h, the PVDF membrane was incubated overnight with primary antibody at 4 °C and then with HRP-conjugated secondary antibody at room temperature for 2 h. The following primary antibodies were used: rabbit anti-Alix (Santa Cruz, CA, USA) and rabbit anti-TSG101 (HuaBio, Hangzhou, China).

### Quantitative real-time PCR

Total RNA was extracted from the cells or tissues using TRIzol (Invitrogen, Grand Island, NY) according to instructions supplied by the manufacturer. Then, 1 μg RNA was reverse transcribed into cDNA using the first-strand cDNA Synthesis System (Roche, Nutley, USA). Real-time PCR was performed on the Opticon® System (Bio-Rad, Hercules, CA) using Hieff™ qPCR SYBR® Green Master Mix (No Rox) (Yeasen, Shanghai, China) in 20 μl reaction volumes. The 2^−ΔΔCt^ method was used to analyze the relative gene expression. The primer sequences used in this study are listed in Suppl. Table 1.

### Scratch wound healing assay

To evaluate the ability of EVs to promote HUVEC and HK-2 cell migration, we performed a scratch wound healing test. Cells were seeded into a 24-well plate with complete medium. When cells reached 90% confluence, scratches were made with sterile plastic 10 μl-micropipette tip and replaced with serum-free medium, then EVs or PBS were added to the plate. Under an inverted microscope (Olympus, Lake Success, NY), at least five fields of view images were taken for each scratch at 0, 12, and 24 h, then the movement distance was quantified using the ImageJ software.

### Tube formation assay

To detect the angiogenic effect of EVs, we performed a tube formation assay. Each well was added 200 μl of Matrigel to coat the 48-well plate and incubated at 37 °C for 30 min. After the Matrigel solidified, 5 × 10^4^ cells per well were added to the solidified Matrigel, then EVs or PBS was added, and the cells were incubated at 37 °C for 6 h. The tube formation was quantitatively analyzed with ImageJ software.

### Detection of cellular reactive oxygen species

The cells were placed in a 24-well plate (2 × 10^4^ cells per well). After the cells were attached to the wall, HK-2 cells were treated with 500 μM concentration of H_2_O_2_ for 3 h to simulate the damage of renal tubular epithelial cells caused by ischemia-reperfusion in vitro. After H_2_O_2_ was removed, the cells were cultured in medium containing EVs at 37 °C and 5% CO_2_ for 48 h. HK-2 cells were washed with PBS for 3 times, then 10 μm DCFH-DA (Beyotime, Shanghai, China) was added and incubated at 37 °C in darkness for 20 min. Then the cells were washed with PBS for 3 times to fully remove the DCFH-DA that did not enter the cells. Fluorescence intensity was recorded using a fluorescence microscope (Olympus, Japan).

### Statistical analysis

All presented results were obtained from at least three independent experiments for each condition. Data are expressed as mean ± SEM. Statistical analyses were performed by one- or two-way analysis of variance using GraphPad Prism software. Differences were considered statistically significant at p < 0.05.

## Results

### Characterization of extracellular vesicles derived from ESC and MEF

**Fig. 1:**
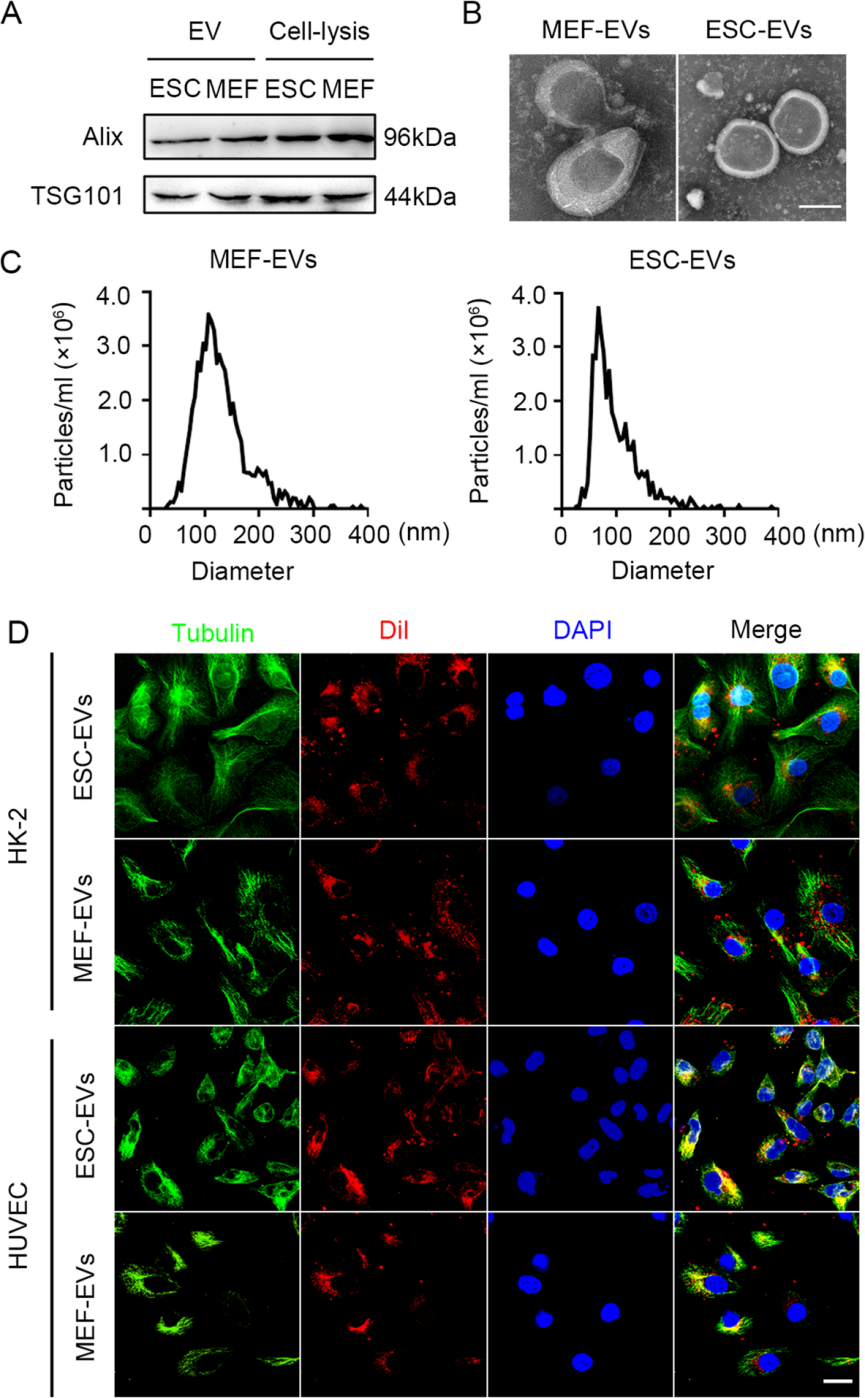
Characterization of extracellular vesicles derived from ESC and MEF. **a** Western blot analysis of the marker of EVs, Alix, and TSG101, in ESC-EVs, MEF-EVs, ESC, and MEF. **b** Transmission electron microscope (TEM) image of MEF-EVs and ESC-EVs. Scale bar represents 50 nm. **c** Nanoparticle tracking analysis (NTA) indicated the particle size distribution of MEF-EVs and ESC-EVs. **d** Internalization of ESC-EVs and MEF-EVs were analyzed by immunofluorescence detection. DiI-labeled EVs (red) were detected in the HK-2 cells and HUVECs which were labeled by anti-Tubulin (green). Scale bar represents 50 μm

Using ultracentrifugation, ESC-EVs and MEF-EVs were isolated from the supernatant of ESCs and MEF cells, respectively. MEF cells were used here as a control to exclude all influence of non-ESCs-EVs [31]. Western blot showed that ESC-EVs and MEF-EVs and their respective source cells expressed exosomal markers Alix and TSG101 (Fig. 1a). The transmission electron microscope (TEM) image showed that ESC-EVs and MEF-EVs were all round vesicles wrapped in membranes. The size of ESC-EVs is slightly smaller than that of MEF-EVs (Fig. 1b), which was next confirmed by the results of nanoparticle tracking analysis (NTA). NTA showed that the diameters of ESC-EVs and MEF-EVs were ~ 80 nm and ~ 110 nm, respectively (Fig. 1c). To test whether EVs can be internalized into kidney cells, we labeled EVs with DiI (red) and incubated them with Tubulin-labeled human renal tubular epithelial cells (HK-2 cells) for 6 h. The co-localization of Dil-labeled EVs and Tubulin-labeled HK-2 cells was observed by immunofluorescence, indicating that EVs were taken up by HK-2 cells (Fig. 1d). EVs mainly localized at cytoplasm of HK-2 cells. The same results were also obtained in human umbilical vein endothelial cells (HUVEC) (Fig. 1d). The above data indicated that EVs were successfully isolated from ESCs and MEFs and could be effectively internalized into the HK-2 cells and HUVEC.

### ESC-EVs promote recovery of renal function in AKI mice

**Fig. 2:**
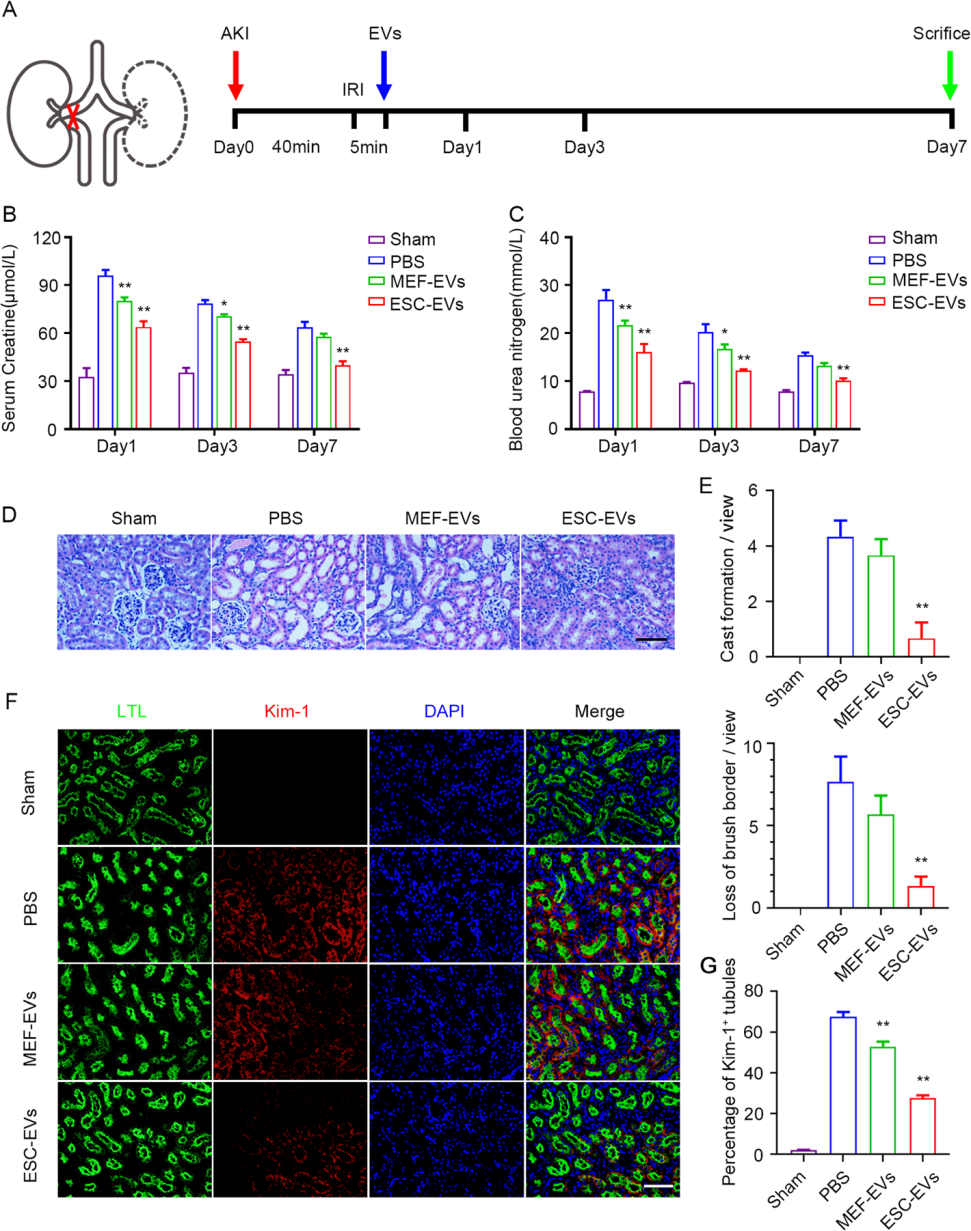
ESC-EVs promote recovery of renal function. **a** Schematic illustration for AKI and EV treatment. The left renal pedicle was clamped with noninvasive microvascular forceps for 40 min and contralateral nephrectomy was performed. After 5 min eperfusion, 100 μg EVs were injected into the upper and lower poles of the left renal cortex. The kidney tissues were collected on days 1, 3, and 7 after the injury. **b** The serum creatinine level of each group was measured on days 1, 3, and 7 after AKI. **c** The blood urea nitrogen level of each group was measured on days 1, 3, and 7 after AKI. **d** Histological analysis of kidney injury by H&E staining at day 3 after AKI. Scale bar represents 100 μm. **e** Quantification of pathological changes in renal tissue, such as cast formation and loss of brush border. Quantitative analysis of 5 random fields in tissue sections of 5 different mice. **f** Representative images of Kim-1 (red) immunofluorescence staining at day 3 after AKI. The proximal tubules were co-stained by FITC-labeled LTL (green). Scale bar represents 100 μm. **g** Quantification of the percentage of Kim-1-positive injured tubules. Quantitative analysis of 5 random fields in tissue sections of 5 different mice. The data are presented as mean ± SEM. (n = 5; ^*^P < 0.05 versus PBS, ^**^P < 0.01 versus PBS)

To determine whether ESC-EV treatment could promote renal recovery after AKI in mice, the left renal pedicle was clamped with noninvasive microvascular forceps for 40 min and contralateral nephrectomy was performed. After 5 min reperfusion, 100 μg EVs were injected into the upper and lower poles of the left renal cortex (Fig. 2a). We used histological analysis and renal function analysis to explore the efficacy of ESC-EVs in the treatment of AKI, and MEF-EVs served as a control. BUN (blood urea nitrogen) and Scr (serum creatinine) are common indicators of renal function, which are all filtered through the glomerulus. When the functions of glomerular were impaired, the concentrations of BUN and Scr increase significantly [41]. So we tested the concentrations of BUN and Scr in the serum at days 1, 3, and 7 after AKI to assess renal function. After AKI, the level of BUN and Scr increased significantly in the PBS group compared with sham, suggesting the kidney was damaged. ESC-EV treatment significantly reduced BUN and Scr levels compared with PBS group, suggesting that ESC-EVs improved glomerular filtration function (Fig. 2b, c). At day 3 (early stage after AKI), we evaluated the histological structure of the kidneys in different groups by HE staining. In PBS group, we could see the typical characteristics of renal ischemia-reperfusion, including cast formation, brush border loss, and the number of necrotic tubules increasing significantly. These pathological phenotypes were significantly recovered in the group of ESC-EV treatment, but not in the MEF-EVs group (Fig. 2d). The cast formation and the loss of brush border in different groups were analyzed statistically (Fig. 2e). AT day 3 after AKI, kidney injury marker (Kim-1), an outstanding indicator of kidney injury [42, 43], was upregulated in PBS group by immunofluorescence staining. In the group of ESC-EV treatment, the expression of Kim-1 has been greatly reduced compared with that in the PBS and MEF-EVs groups (Fig. 2f). We counted the Kim-1^+^ tubules in each sight field, and the results showed that compared with 70% Kim-1^+^ tubules in the PBS group, it was only about 30% in the ESC-EVs group, indicating ESC-EV treatment promoted the recovery of kidney functions (Fig. 2g). Lotus tetragonolobus lectin (LTL)-positive tubules were partially restored compared to the injured kidney after ESC-EV treatment (Fig. 2g). We also examined the effects of EVs to other major organs, including heart, liver, spleen, lung, and intestine. Histological analysis found no significant structural differences in these organs after EV treatment, suggesting that EVs are not toxic in vivo (Figure S1). In conclusion, ESC-EVs promoted physiological repair after AKI, to restore normal renal structure and function.

### ESC-EVs ameliorate fibrosis of the kidney after AKI

After AKI, the inflammatory cascade caused by various pathological mechanisms in the acute phase leads to progressive impairment of kidney function. In this case, the body changes adaptively and the disease enters the chronic phase, a process known as pathological repair [44]. During this process, renal tubular epithelial cells exhibit senescent phenotypes, such as cell cycle arrest and cell proliferation slowing down [45]. This accumulation of pathological repair leads to renal fibrosis and eventually to CKD or ESRD.

**Fig. 3:**
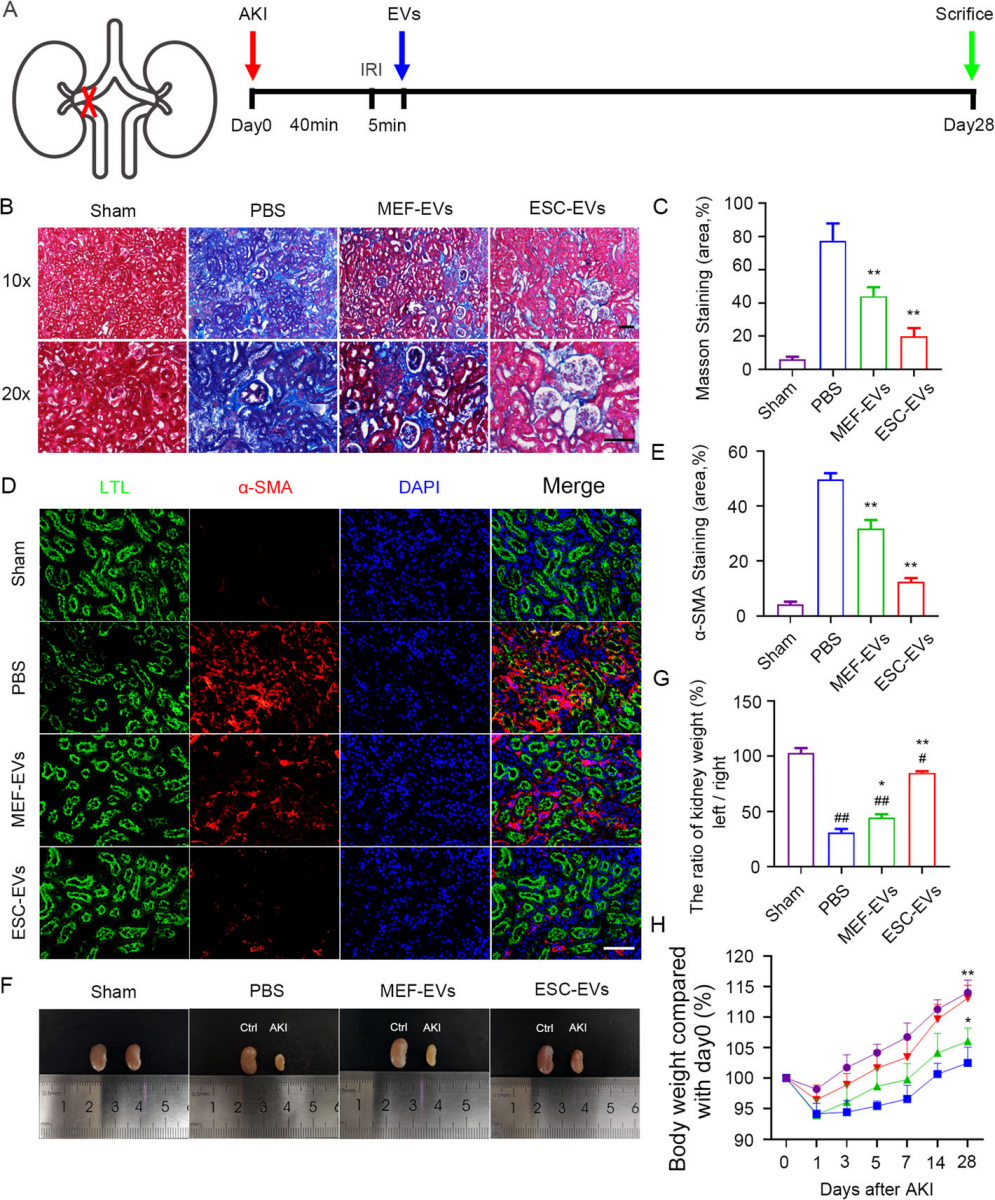
Treatment of ESC-EVs inhibit renal fibrosis. **a** Schematic illustration for AKI and EV treatment. The left renal pedicle was clamped with noninvasive microvascular forceps for 40 min without any operation on the contralateral. After 5 min eperfusion, 100 μg EVs were injected into the upper and lower poles of the left renal cortex. The kidney tissues were collected on day 28 after the injury. **b** Representative images of Masson staining for renal tissues harvested on day 28 post AKI. Scale bar represents 100 μm. **c** Quantification of the area of Masson staining. Quantitative analysis of 5 random fields in tissue sections of 5 different mice. **d** Representative images of α-SMA immunofluorescence staining at day 28 after AKI. Scale bar represents 100 μm. **e** Quantification of the area of α-SMA staining. Quantitative analysis of 5 random fields in tissue sections of 5 different mice. **f** Morphology of the left and right kidneys of mice after 28 days of AKI. The kidney on the left is the right kidney of the mouse (control side), and the right is the left kidney of the mouse (ischemia-reperfusion side). The two kidneys in each photo were isolated from same mouse. **g** The percentage of the weight of the left kidney (ischemia-reperfusion side) versus the weight of the right kidney (control side) at day 28 after AKI. The data are presented as mean ± SEM. **h** Changes in body weight of mice (vs. 0 days after AKI) over time. The data are presented as mean ± SEM. (n = 5; ^*#*^*P* < 0.05 versus Sham, ^*##*^*P* < 0.01 versus Sham, ^*^P < 0.05 versus PBS, ^**^P < 0.01 versus PBS)

To test the functions of ESC-EVs in the later pathological repair, we established unilateral kidney injury mice model (Fig. 3a). Using this model, we firstly evaluated the level of renal fibrosis in different groups by Masson trichrome staining, and the quantitative statistics of fibrosis area showed that ESC-EVs significantly alleviated fibrosis compared with other groups (Fig. 3b, c). Myofibroblasts are the main cause of renal interstitial fibrosis by secreting collagen [46]. We next evaluated the level of renal fibrosis by immunostaining of α-SMA (alpha-smooth muscle actin), the best marker for myofibroblasts. The decrease of α-SMA in ESC-EV treatment group also indicated that ESC-EVs can alleviate renal fibrosis, which was consistent with the results of Masson staining (Fig. 3d, e).

With the continuous development of renal fibrosis, healthy nephrons are gradually lost, which in turn causes renal atrophy [46]. We compared the volume and weight of the injured kidney (the left kidney of the mouse) and the uninjured kidney (the right kidney of the same mouse) in each group at 28 days after AKI, and found that the injured kidney was obviously smaller than that of uninjured kidney in the PBS group, suggesting the injured kidney without any treatment has atrophy. However, with ESC-EV treatment, the renal atrophy was alleviated significantly. MEF-EV treatment could partially inhibit the atrophy, but which was far less effective than ESC-EVs (Fig. 3f, g). Besides, the average body weight of mice treated with ESC-EVs was much higher than that of two control groups, and similar to that of the sham (Fig. 3h). These data indicated that the treatment of ESC-EVs could reduce renal fibrosis and promote the recovery of renal function.

### ESC-EVs promote angiogenesis in vivo and vitro after AKI

**Fig. 4:**
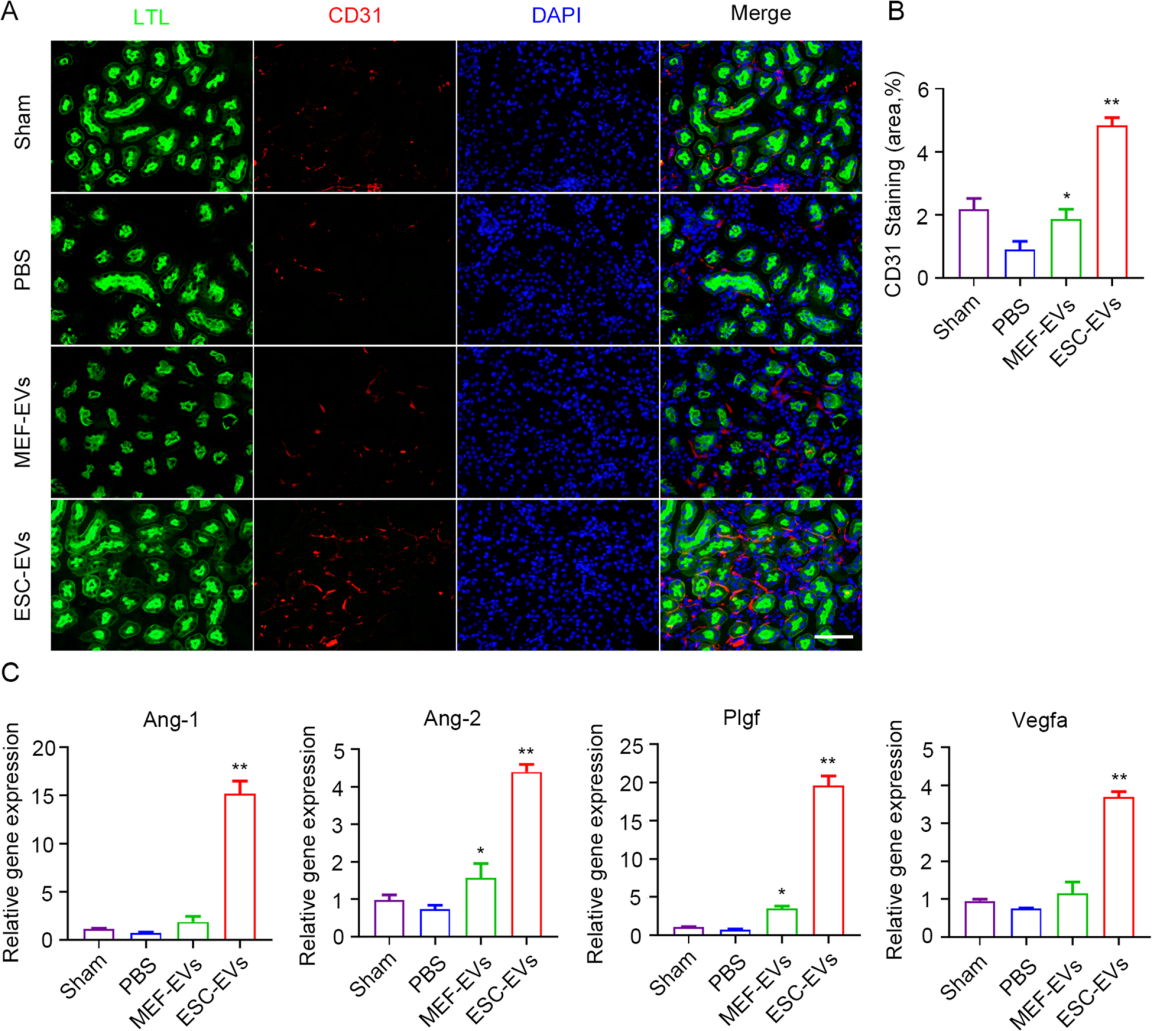
ESC-EVs have the effect of promoting angiogenesis in vivo. **a** Representative images of CD31 immunofluorescence staining at day 14 after AKI. Scale bar represents 100 μm. **b** Quantification of the area of CD31 staining. Quantitative analysis of 5 random fields in tissue sections of 5 different mice. **c** Real-time qPCR analysis of angiogenic factor-related genes in injured kidneys at day 14 after AKI. The data are presented as mean ± SEM. (n = 5; ^*^P < 0.05 versus PBS, ^**^P < 0.01 versus PBS)

Studies have shown that tissue hypoxia caused by capillary reduction is closely related to organ fibrosis [47]. So we next explored whether ESC-EV treatment could recover peritubular capillary density after AKI. CD31, a marker of vascular endothelial cells [48, 49], immunofluorescence staining was performed on the kidney tissue of mice on day 14 after AKI. We observed that the peritubular capillary density of mice decreased significantly after AKI, which was rescued by ESC-EV treatment, indicating that ESC-EVs could promote angiogenesis in vivo (Fig. 4a, b). RT-PCR was used to detect the expression of proangiogenic-related genes in kidney tissues of different groups of mice [50]. We found that with the treatment of ESC-EVs the expression of proangiogenic genes (*Ang-1*, *Ang-2*, *Plgf*, and *Vegfa*) were increased (Fig. 4c). These data suggested that ESC-EVs promoted angiogenesis in vivo.

**Fig. 5:**
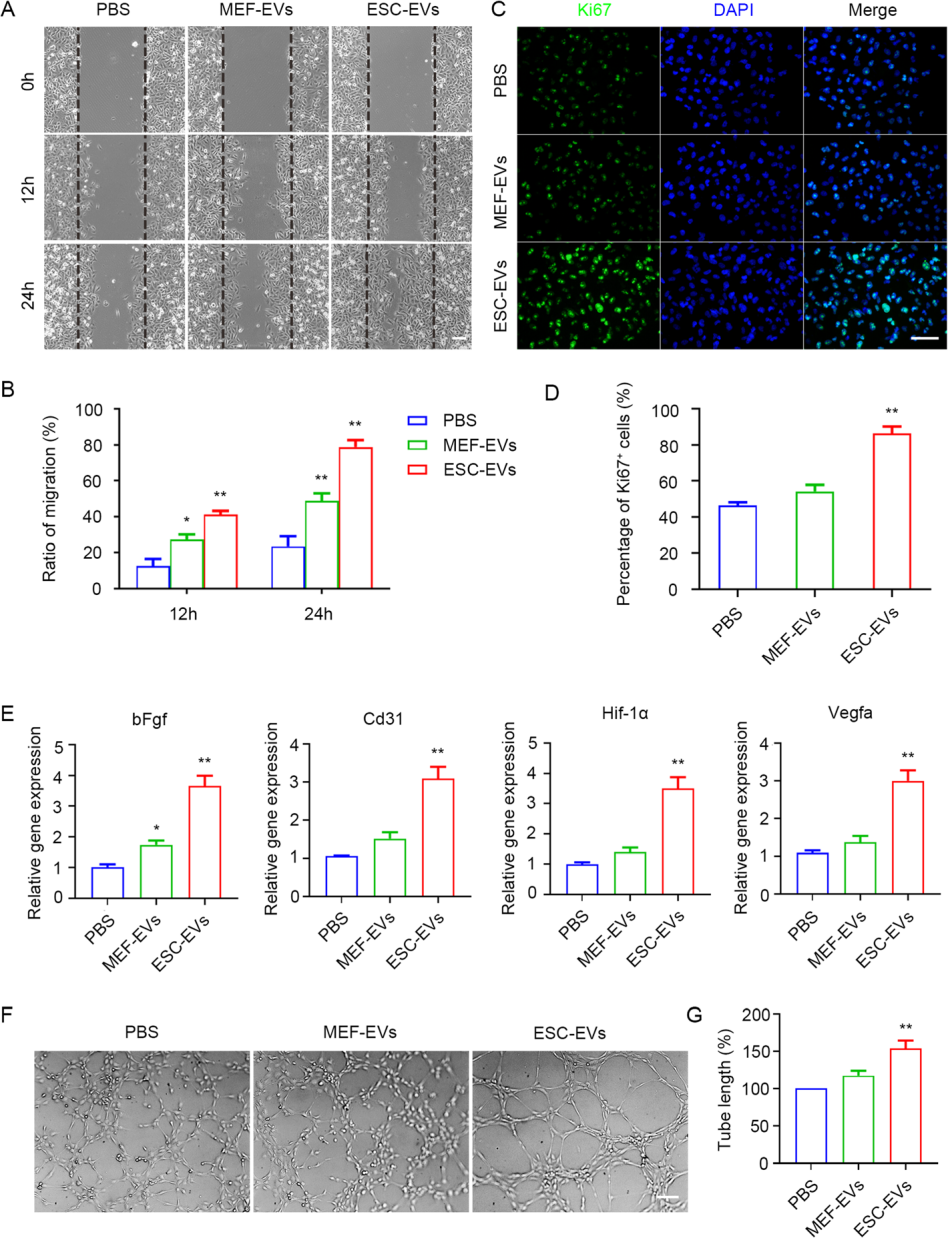
ESC-EVs also promote angiogenesis in vitro. **a** Scratch assay of HUVECs treated with EVs. Scale bar represents 100 μm. **b** Quantification of the migration ratio of HUVECs in each group. **c** Immunostaining analysis for Ki67 in HUVECs treated with EVs for 24 h. Scale bar represents 100 μm. **d** Quantification of Ki67-positive HUVECs in each group. **e** Real-time qPCR analysis of angiogenic factor-related genes in HUVECs treated with EVs for 24 h. **f** Optical images of tube formation of HUVECs in each group. Scale bar represents 100 μm. **g** Quantitative evaluation of the tube length in each group. The data are presented as mean ± SEM. (n = 3; ^*^P < 0.05 versus PBS, ^**^P < 0.01 versus PBS)

Next, we used HUVECs as a model to explore the proangiogenesis effect of ESC-EVs. First, cell counting kit-8 (CCK-8) experiments showed that ESC-EVs promoted the proliferation of HUVECs in a dose-dependent manner, with a peak of 100 μg/ml (Figure S2A). Therefore, in the following experiments, HUVECs were treated with EVs with a concentration of 100 μg/ml for 24 h. The wound healing assay showed that ESC-EVs promoted HUVEC migration (Fig. 5a), and the migration rate in different groups of cells was statistically analyzed (Fig. 5b). Ki67 immunofluorescence staining and quantitative statistical analysis showed that ESC-EVs promoted HUVEC proliferation in vitro (Fig. 5c, d). RT-PCR analysis indicated that ESC-EVs promoted the expression of proangiogenic-related genes bFgf, Cd31, Hif-1α, and Vegfa in HUVECs (Fig. 5e). To further investigate the proangiogenic effect of the ESC-EVs, we conducted the tube formation assay in vitro. We found that when HUVECs were cultured in Matrigel-coated plates, they formed tubular structures that connected to form a network of blood vessels. When treated HUVECs with ESC-EVs, the number and length of tubules increased significantly, suggesting ESC-EVs significantly promoted HUVECs to form microvessels (Fig. 5f). We performed a statistical analysis of tubule length in different groups (Fig. 5g). These data suggested that ESC-EVs promote angiogenesis in vivo and in vitro, which might contribute to the reduction of renal fibrosis.

### ESC-EVs promote recovery of the physiological function of renal tubular epithelial cell

**Fig. 6:**
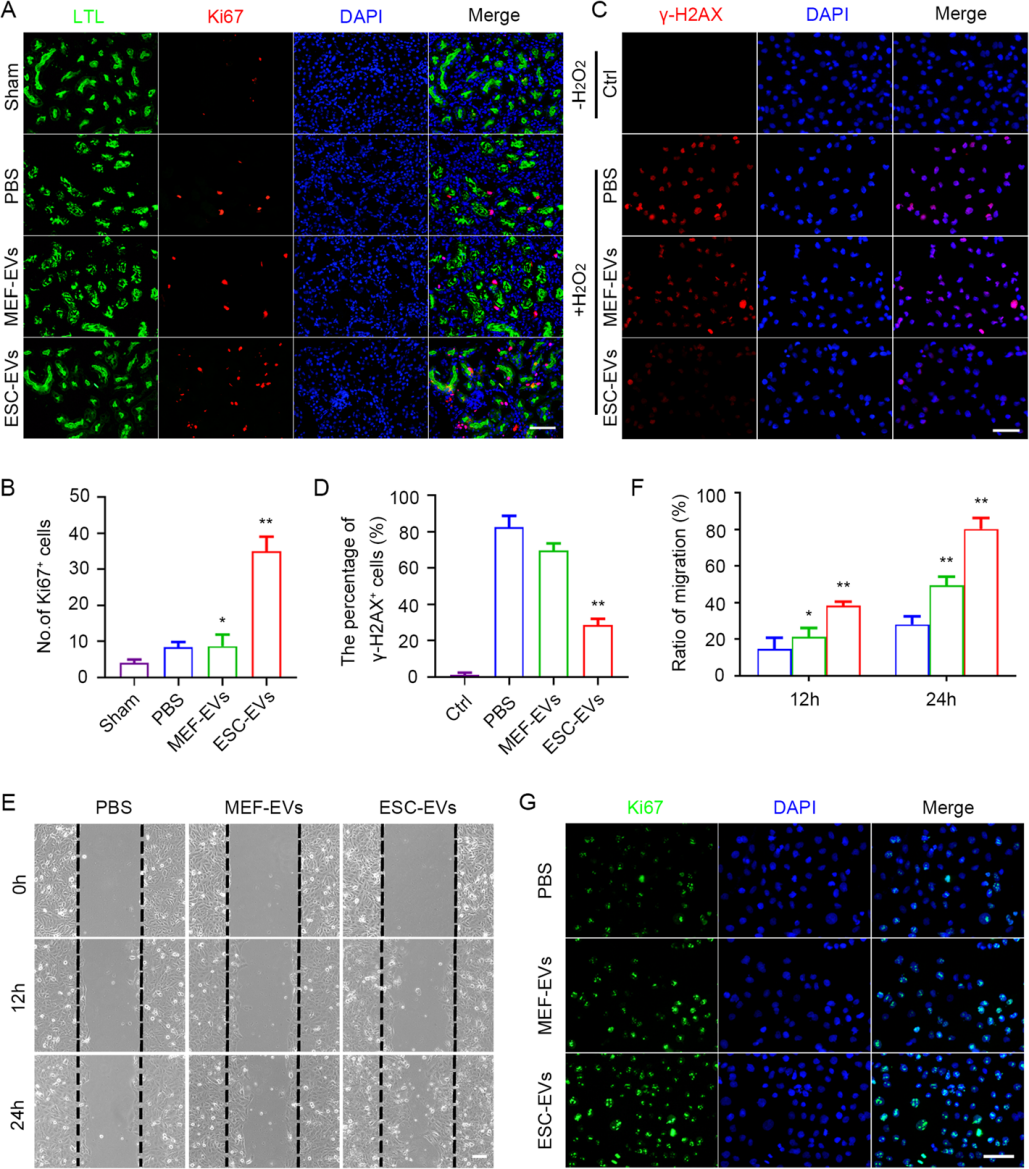
ESC-EV treatment enhances the physiological function of renal tubular epithelial cells. **a** Representative images show Ki-67 immunostaining (red) detected in renal tubular epithelial cells (LTL, green) at day 3 after AKI. Scale bar represents 100 μm. **b** Quantitative analysis of Ki-67 immunostaining at day 3 after AKI. Quantitative analysis of 5 random fields in tissue sections of 5 different mice. **c** Representative immunofluorescence images of γ-H2AX expression in HK-2 cells with and without H_2_O_2_ pretreatment. Scale bar represents 100 μm. **d** Quantification of the percentage of γ-H2AX-positive HK-2 cells. Quantitative analysis of 5 random fields in tissue sections of 5 different mice. **e** Scratch assay of HK-2 cells treated with EVs. Scale bar represents 100 μm. **f** Quantification of the migration ratio of HK-2 cells in each group. **g** Immunostaining analysis for Ki67 in HK-2 cells treated with EVs for 24 h. Scale bar represents 100 μm. The data are presented as mean ± SEM. (n = 5; ^*^P < 0.05 versus PBS, ^**^P < 0.01 versus PBS)

In the early stages after AKI, the viable renal tubular epithelial cells remaining after injury dedifferentiate, proliferate, migrate to the injured site, and then differentiate into mature renal tubular cells to replace the originally damaged cells and restore the tubule function [51]. We next wondered whether ESC-EVs could promote the physiological repair process after AKI by promoting the proliferation of renal tubular epithelial cells that survived the injury. First, we performed Ki67 immunofluorescence staining on day 3 after AKI, it was observed that the number of Ki67-positive cells increased significantly in the ESC-EV treatment group, and most of the Ki67-positive cells were located in renal tubules (Fig. 6a). The number of Ki67^+^ cells in ESC-EV treatment groups was more than the other two groups (Fig. 6b). This suggested that ESC-EV treatment promote the proliferation of renal tubular epithelial cells, which contributed to physiological repair after AKI. To further explore the physiological effects of ESC-EVs on renal tubular epithelial cells, we directly used HK-2 cells (human renal tubular epithelial cells) to investigate the effect of ESC-EVs in vitro. CCK-8 assay showed that the optimal concentration of ESC-EVs for HK-2 cells was 100 μg/ml (Figure S2B). Firstly, HK-2 cells were treated with H_2_O_2_ at a concentration of 500 μM for 3 h and then replaced with normal medium without H_2_O_2_ for further culture for 48 h to simulate the damage of renal tubular epithelial cells caused by ischemia-reperfusion in vitro. As shown in Fig. 6c, after the treatment of H_2_O_2_, DNA damage was obvious indicated by γ-H2AX immunofluorescence staining. DNA damage induced by H_2_O_2_ could be rescued by ESC-EV treatment, we also found that ESC-EVs inhibited the increase of intracellular reactive oxygen species (ROS) after H_2_O_2_ stimulation (Figure S3A, B), suggesting that ESC-EVs protected renal tubular epithelial cells from oxidative stress damage (Fig. 6d). Furthermore, ESC-EVs also promoted HK-2 cell migration (Fig. 6e, f) and proliferation in vitro (Fig. 6g). All these findings indicated that ESC-EVs could rescue the physiological function of renal tubular epithelial cells by protecting cells from oxidative stress and promoting proliferation.

### ESC-EVs activate Sox9 expression to promote kidney repair after AKI

**Fig. 7:**
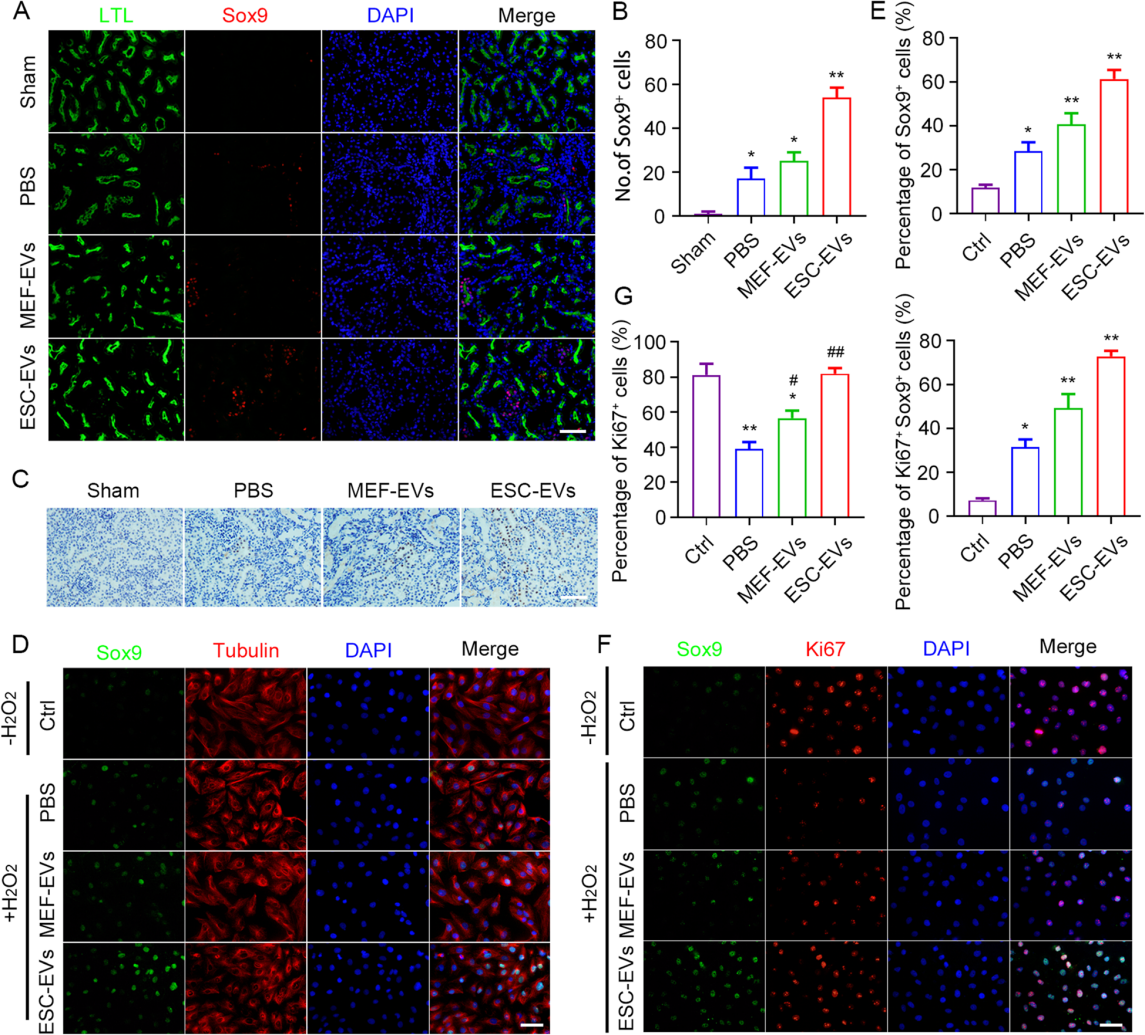
ESC-EV treatment activate Sox9 expression in the injured kidney. **a** Representative images of Sox9 immunofluorescence staining at day 3 after AKI. Scale bar represents 100 μm. **b** Quantification of Sox9-positive cells in each group. **c** Representative images of Sox9 immunohistochemical staining at day 3 after AKI. Scale bar represents 100 μm. Quantitative analysis of 5 random fields in tissue sections of 5 different mice. **d** Representative immunofluorescence images of Sox9 expression in HK-2 cells with and without H_2_O_2_ pretreatment. Scale bar represents 100 μm. **e** Quantification of the percentage of Sox9-positive HK-2 cells. **f** Representative immunofluorescence images of Sox9 and Ki67 expression in HK-2 cells with and without H_2_O_2_ pretreatment. Scale bar represents 100 μm. **g** Quantification of the percentage of Ki67-positive HK-2 cells and Ki67/Sox9 double-positive HK-2 cells. The data are presented as mean ± SEM. (n = 5; ^*^P < 0.05 versus Ctrl, ^**^P < 0.01 versus Ctrl, ^*#*^*P* < 0.05 versus PBS, ^*##*^*P* < 0.01 versus PBS)

Studies have shown that Sox9 plays an important role in the formation and regeneration of renal tubular epithelial cells and is critical in the development of human and mouse renal embryos [52, 53]. The activation of Sox9^+^ cells is a key renal regeneration response, and the lineage tracing showed that the descendants of Sox9^+^ cells regenerated tubular epithelial cells [52, 54]. We found that Sox9^+^ cells were activated after injury, and the number of Sox9^+^ cells was significantly increased after ESC-EV treatment by immunofluorescence staining (Fig. 7a, b), which was then confirmed by immunohistochemistry (Fig. 7c). Sox9 was mainly expressed in renal tubular epithelial cells. Therefore, we used HK-2 cells to further investigate the Sox9 activation effect of ESC-EVs in vitro. Similarly, we found that hypoxia injury induced by H_2_O_2_ activated the expression of Sox9 in HK-2 cells, and ESC-EV treatment further upregulate the expression of Sox9, which was consistent with the results obtained in vivo (Fig. 7d, e). Most Sox9^+^ cells were also positive for Ki67 (Fig. 7f, g), indicating that Sox9^+^ cells were in a proliferative state, which may contribute to the regeneration of the damaged renal tubular epithelial cells and the restoration of the physiological function of the damaged kidney. The above results indicated that ESC-EVs facilitate regenerate damaged renal tubular epithelial cells by activating Sox9^+^ cells.

## Discussion

**Fig. 8:**
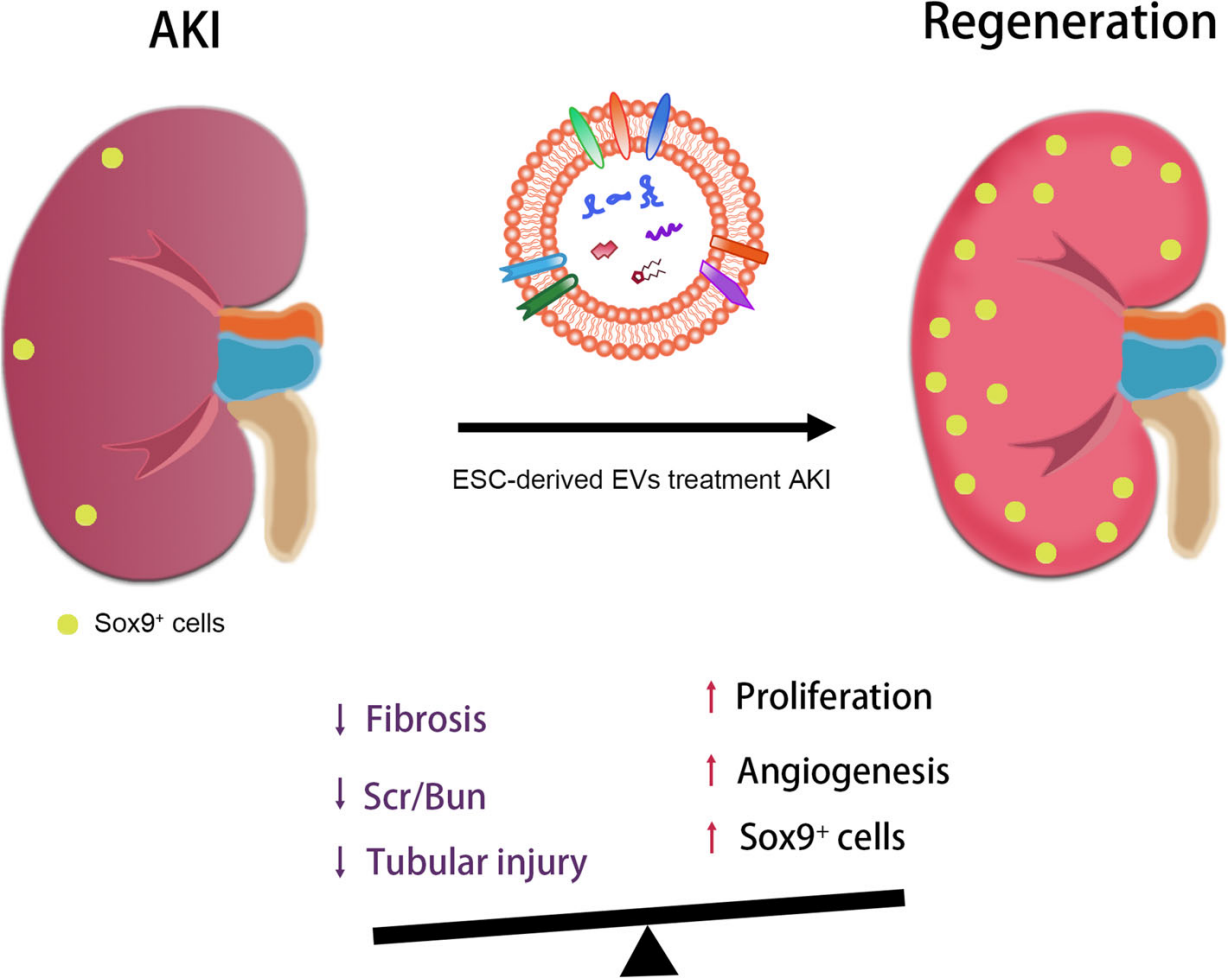
Schematic diagram of ESC-EVs promote AKI healing by activating Sox9^+^ cells. ESC-EV treatment inhibits renal fibrosis, renal tubular injury, and the increase of Scr and BUN levels after AKI. It promotes the proliferation of renal tubular epithelial cells, injured renal angiogenesis, the expression of Sox9 and accelerates the proliferation of Sox9^+^ cells. The above approaches promote the physiological repair process, inhibit the pathological repair process of the kidney after AKI, and restore the structure and function of the injured kidney

In the present study, our results showed that ESCs derived EVs alleviated murine AKI by promoting the physiological repair and inhibiting the pathological repair process. In addition, the treatment of ESC-EVs activated Sox9^+^ cells in the damaged kidney. We successfully isolated and identified EVs from ESCs and MEF. The EVs we extracted can be effectively internalized into renal cells and were biocompatible. ESC-EVs significantly promoted the recovery of renal structure and function, angiogenesis, and proliferation of renal tubular epithelial cells. Meantime, ESC-EV treatment inhibited renal fibrosis after AKI and the damage to renal tubular epithelial cells caused by hypoxia. ESC-EVs promoted physiological repair after AKI and inhibit pathological repair, allowing the damaged kidney to recover its structure and function (Fig. 8).

ESCs are derived from the inner cell mass of preimplantation embryos. They have the characteristics of infinite proliferation, self-renewal, and pluripotent and can be induced to differentiate into all derivatives of the three primary germ layers: ectoderm, endoderm, and mesoderm [55]. ESCs have promising prospects in tissue and cell production, cell therapy, and so on [56–59]. However, the application of ESCs in clinical practice is limited by the problems of immune rejection, tumorigenicity, and ethics [13, 28, 60]. In order to avoid these restrictions, many efforts have been tried. Studies have shown that the conditioned medium derived from ESCs can promote cell proliferation and tissue regeneration, which is mediated by the cytokines secreted by cells [56]. EVs secreted by cells containing a variety of RNA and proteins and have low immunogenicity [61], have a wide range of therapeutic effects and have received extensive attention in recent years [62–67]. EVs derived from ESCs can enhance endogenous repair of myocardial injury, ischemia-reperfusion repair, and promote the therapeutic effect of MSCs [31, 33, 68, 69]. The functions of EVs, including anti-apoptosis, promotion of angiogenesis, and anti-fibrosis, dependent on the cells from which they are derived [70, 71]. Here, we found that ESC-EVs facilitate the recovery of renal structure and function after AKI, which expands our understanding of ESC-EVs, especially for the therapeutic effect of AKI.

AKI is characterized by a sudden decrease in glomerular filtration rate and a sharp decrease in renal function and is one of the common clinical critical diseases [72]. The repair process of AKI includes physiological and pathological repair [73]. When the damage factor is severe or persistent, the disease enters the chronic phase, a process known as pathological repair. The accumulation of pathological repair leads to renal fibrosis, which eventually develops into CKD or ESRD. Hypoxic injury has been shown to lead to a decrease in peritubular capillaries, which limits the supply of oxygen to the interstitium, leading to tubule atrophy and fibrosis. Excessive collagen further reduces the blood flow around the renal tubules and aggravates the hypoxia of the renal tubules and the shedding of the nephron, entering a self-reinforcing vicious circle, accelerating the progression of renal diseases [46, 47, 74, 75]. Therefore, tissue hypoxia caused by decreased capillaries is closely related to organ fibrosis. In this study, we found that ESC-EVs promoted physiological repair by promoting the proliferation of renal tubular epithelial cells after AKI. ESC-EVs also inhibited renal fibrosis by promoting angiogenesis, which prevented the pathologic repair process after AKI.

Sox9 plays an important role in the development of many organ systems in mammals [76]. In the kidney, Sox9 deficiency leads to hydronephrosis and renal hypoplasia. It was found that the deletion of Sox8 and Sox9 during renal embryonic development led to renal hypoplasia in mice [77]. Kumar et al. identified Sox9 activation as an early response to AKI, the lineage tracing of Sox9^+^ cells showed that most of the descendants regenerated into renal tubular epithelial cells [54]. Kang et al. also found that tubule-specific Sox9 deficiency resulted in reduced epithelial cell proliferation, increased fibrosis, and more severe renal injury [53]. Our study found that ESC-EV treatment could significantly increase the number of Sox9^+^ cells in the renal tubular epithelial cells after AKI, suggesting that ESC-EVs regenerate damaged renal tubular epithelial cells by activating Sox9^+^ cells, but the mechanism of Sox9^+^ cells activated after AKI in renal repair remain unclear and need further study. Research have reported that the expression of Sox9 is regulated by Wnt / β-catenin signaling [78], and the Wnt signaling pathway undergoes redox-dependent regulation [79, 80]. Ultimately, ESC-EVs promote renal repair after AKI may through resistance to oxidative stress.

The regenerative ability of mesenchymal stem cell-derived-EVs (MSC-EVs) on AKI has been demonstrated before [52, 81–84]. Bruno et al. reported that MSC-EVs accelerated the glycerol-induced morphological and functional recovery of AKI in mice by inducing renal tubular cell proliferation and found that MSC-EVs activated the proliferative program in renal tubular cells that survived injury via a horizontal transfer of mRNA [85]. Currently, the treatment of AKI by EVs mainly focused on MSC-EVs, and the effect of ESC-EVs on renal repair after AKI has not been reported. Our data indicate that ESC-EVs facilitate the repair of injured kidney by promoting physiological repair and inhibiting the pathological repair process. This is the first time to report the therapeutic effect of ESC-EVs on AKI.

Together, our data shows the great potential of ESC-EVs in clinical treatment. However, before ESC-EVs can be successfully used in clinic, more detailed research is needed, including clarifying its potential mechanisms and exploring ways to improve its efficiency. Oxidative stress plays a key role in the pathogenesis of sepsis and ischemic acute kidney injury, because kidney tissue is rich in angiogenesis and continuous metabolic activity and is particularly vulnerable to the harmful effects of ROS [86, 87]. Excessive ROS produced by ischemia and reperfusion are activators of tissue damage [88]. ROS promote vascular dysfunction, inflammation, and renal tubular cytotoxicity [86, 89]. Patients with acute renal failure (ARF) accumulate features of oxidative stress, such as lipid peroxidation markers, deoxyribonucleic acid oxidation products, and advanced glyc (oxid) ation end products, and they have a high mortality rate [90]. Himmelfarb et al. found that oxidative stress increased in ARF patients by detecting the oxidation levels of plasma protein in ARF patients, healthy subjects, and critically ill patients, suggesting that the use of antioxidant therapy may reduce the mortality of the patient population [91]. A large number of studies have shown that the use of endogenous antioxidants can directly or indirectly prevent the production of ROS and other oxidant sources and reduce the oxidative damage of the kidney, thus promoting the recovery of renal function [92–95]. In this study, we also found that ESC-EVs can inhibit the oxidative stress response, which play an important role in promoting the repair of injured kidney. Moreover, several studies have reported recently that EVs derived from stem cells cultured in hypoxia have greater therapeutic potential in tissue repair than that derived from stem cells cultured in normal condition [96]. It is another potential strategy to improve the therapeutic effects of ESC-EVs in the future.

## Conclusions

In summary, this study explored the effect of ESC-EVs on renal recovery by establishing the AKI model in mice. We found that ESC-EVs promoted the physiological repair of the kidney after AKI and inhibited the pathological repair process, which promotes the restoration of normal structure and function of the injured kidney. This study showed the effectiveness of ESC-EVs in the treatment of AKI and provided a novel strategy for the treatment of AKI, which had great practical value.

## Supplementary Information

**Additional file 1.**

## Data Availability

The dataset used and/or analyzed during the current study are available from the corresponding author upon reasonable request.

## Abbreviations

ESC: Embryonic stem cell
MEF: Mouse embryonic fibroblast
EVs: Extracellular vesicles
ESC-EVs: Embryonic stem cell-derived extracellular vesicles
MEF-EVs: Mouse embryonic fibroblast-derived extracellular vesicles
AKI: Acute kidney injury
CKD: Chronic kidney disease
ESRD: End-stage renal disease
HK-2: Human renal tubular epithelial cell
HUVEC: Human umbilical vein endothelial cell
BUN: Blood urea nitrogen
Scr: Serum creatinine
Kim-1: Kidney injury marker
LTL: Lotus tetragonolobus lectin
α-SMA: Alpha-smooth muscle actin
CCK-8: Cell counting kit-8
TEM: Transmission electron microscopy
DAPI: 4,6-Diamidino-2-phenylindole
ROS: Reactive oxygen species
ARF: Acute renal failure
