# Supplementary material for: Embryonic stem cell-derived extracellular vesicles promote the recovery of kidney injury
Source: Stem Cell Res Ther. 2021 Jul 2;12:379. doi: 10.1186/s13287-021-02460-0 (PMC8254253; doi:10.1186/s13287-021-02460-0)
Supplement: Supplementary file 1 — Additional file 1. [file 13287_2021_2460_MOESM1_ESM.docx]

**Supporting Information:**

**Embryonic stem cell-derived** **extracellular vesicles promote the recovery of kidney injury**

**Yu, *et al.***

**The Following Files are Included:**

**Supplementary Figures and Legends**

**Supplemental Figure 1**

**Supplemental Figure 2**

**Supplemental Figure 3**

**Supplemental Table 1**

**Supplemental Table 2**

**Supplemental Figures and Legends**

**Figure S1**

**
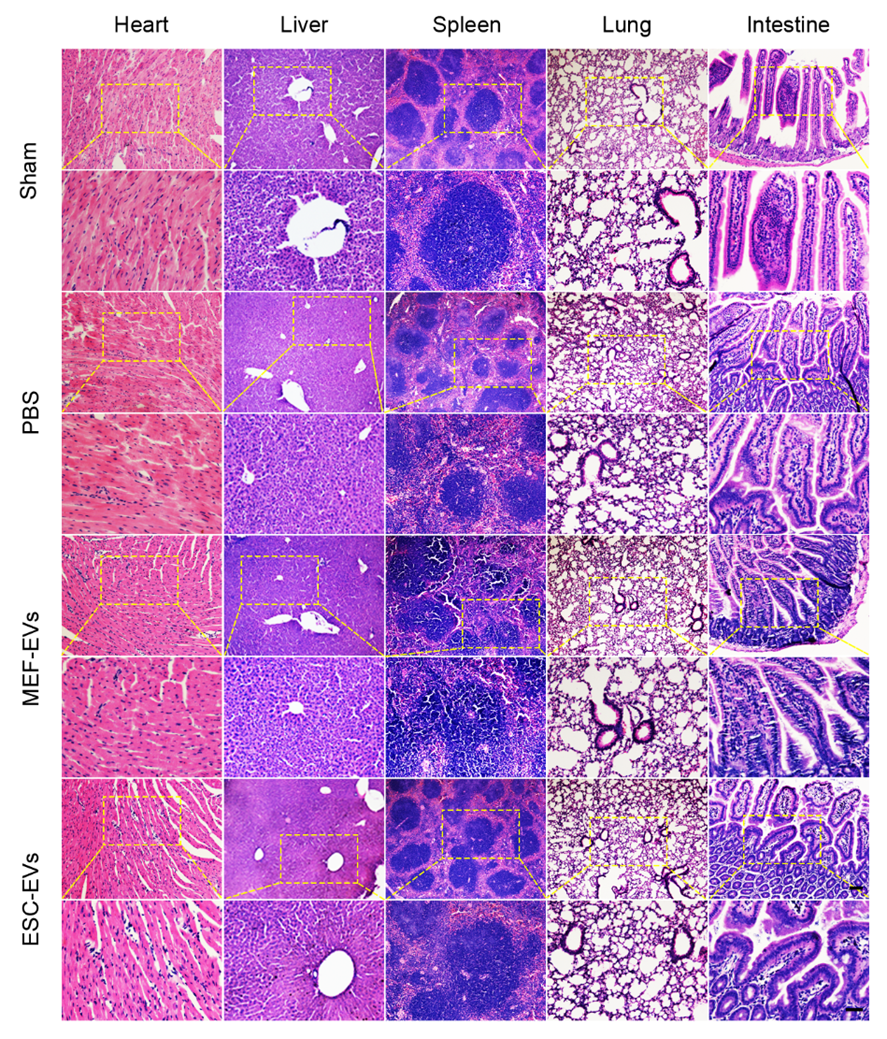
**

**Figure S1** **Assessment of toxicity in EVs treated mice.** HE staining of the heart, liver, spleen, lung, and intestine of mice after 28 days of AKI. Scale bar, 100μm.

**Figure S2**


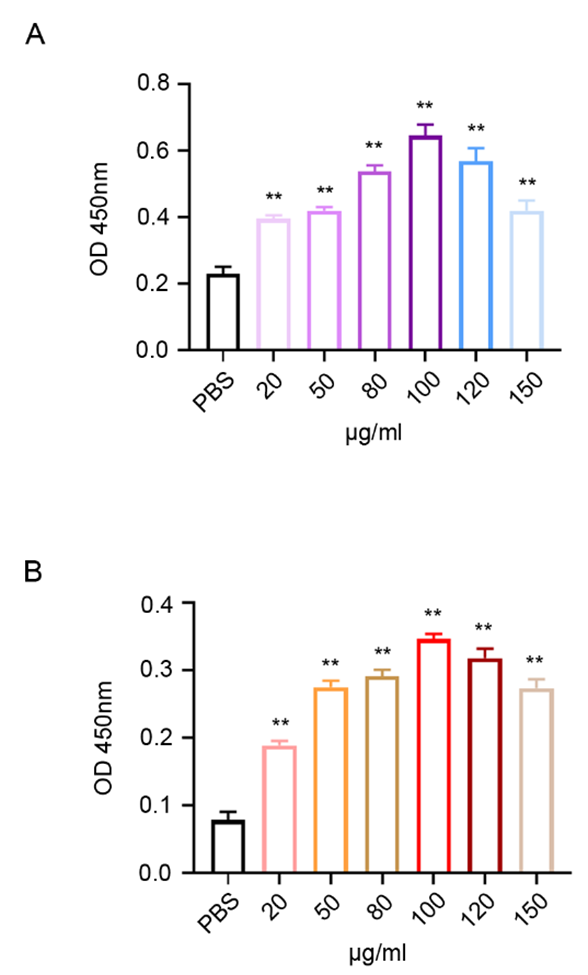


**Figure S2 The optimal concentration of ESC- EVs to promote cell proliferation. A** The optimal concentration of ESC-EVs to promote HUVEC cells proliferation was determined by CCK-8. **B** The optimal concentration of ESC-EVs to promote HK-2 cells proliferation was determined by CCK-8. The data are presented as mean ± SEM. (n = 3; *^**^P* <0.01 versus PBS).

**Figure S3**

**
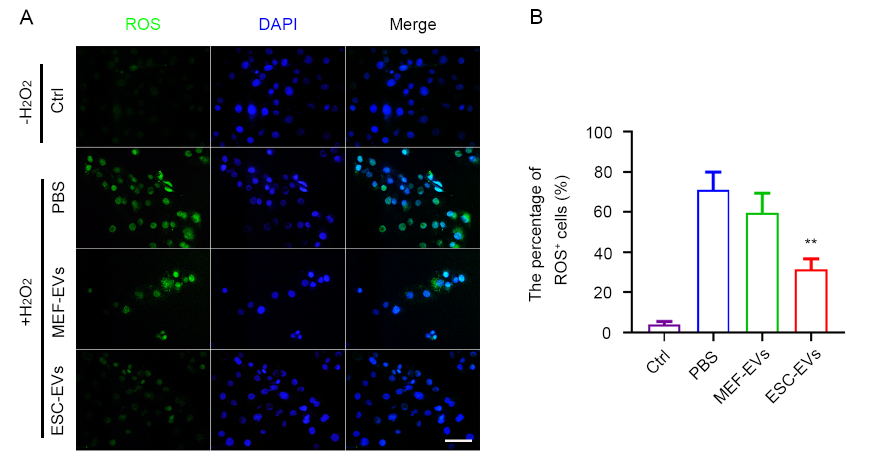
**

**Figure S3 ESC-EVs treatment inhibited the production of intracellular ROS after H_2_O_2_ stimulation. A** Representative immunofluorescence images of ROS in HK-2 cells with and without H_2_O_2_ pretreatment. Scale bar represents 100μm. **B** Quantification of the percentage of ROS positive HK-2 cells.

**Supplemental Tables**

**Table S1. Human primers used for RT-PCR**

| Gene Name | Primers |
| --- | --- |
| GAPDH | Forward: GGAGCGAGATCCCTCCAAAAT  Reverse: GGCTGTTGTCATACTTCTCATGG |
| bFGF | Forward: AGTGTGTGCTAACCGTTACCT  Reverse: ACTGCCCAGTTCGTTTCAGTG |
| CD31 | Forward: CCAAGCCCGAACTGGAATCT  Reverse: CACTGTCCGACTTTGAGGCT |
| HIF-1α | Forward: TTTCCTCAGTCGACACAGCC  Reverse: TCCACCTCTTTTGGCAAGCA |
| VEGFA | Forward: TGTCTAATGCCCTGGAGCCT  Reverse: GTCACATCTGCAAGTACGTTCG |

**Table S2**. **Mouse primers used for RT-PCR**

| Gene Name | Primers |
| --- | --- |
| GAPDH | Forward: AGGTCGGTGTGAACGGATTTG  Reverse: TGTAGACCATGTAGTTGAGGTCA |
| ANG-1 | Forward: CACATAGGGTGCAGCAACCA  Reverse: CGTCGTGTTCTGGAAGAATGA |
| ANG-2 | Forward: CCTCGACTACGACGACTCAGT  Reverse: TCTGCACCACATTCTGTTGGA |
| PLGF | Forward: TCTGCTGGGAACAACTCAACA  Reverse: GTGAGACACCTCATCAGGGTAT |
| VEGFA | Forward: CTGCCGTCCGATTGAGACC  Reverse: CCCCTCCTTGTACCACTGTC |
